# The neutrophil–lymphocyte ratio as a risk factor for all-cause and cardiovascular mortality among individuals with diabetes: evidence from the NHANES 2003–2016

**DOI:** 10.1186/s12933-023-01998-y

**Published:** 2023-09-29

**Authors:** Gaiying Dong, Man Gan, Shilin Xu, Yanlin Xie, Ming Zhou, Liangliang Wu

**Affiliations:** 1 Department of Medical Ultrasound, Guangzhou First People’s Hospital, South China University of Technology, Guangzhou, Guangdong China; 2 Department of Hematology, Guangzhou First People’s Hospital, South China University of Technology, Guangzhou, Guangdong China

**Keywords:** Neutrophil lymphocyte ratio, All-cause mortality, Cardiovascular mortality, Diabetes

## Abstract

**Background:**

Evidence regarding the neutrophil–lymphocyte ratio (NLR) and mortality risk in diabetes patients is scarce. This study investigated the relationship of the NLR with all-cause and cardiovascular mortality risk in diabetes patients.

**Methods:**

Diabetes patients (n = 3251) from seven National Health and Nutrition Examination Survey (NHANES) cycles (2003–2016) were included in this study. The cause of death and mortality status of the participants were obtained from National Death Index records. Restricted cubic spline (RCS) was used to visualize the association of the NLR with mortality risk. The maximally selected rank statistics method (MSRSM) was used to determine the optimal NLR cutoff value corresponding to the most significant association with survival outcomes. Weighted multivariable Cox regression models and subgroup analyses were adopted to assess the association of the NLR with all-cause and cardiovascular mortality. Time-dependent receiver operating characteristic curve (ROC) analysis was conducted to evaluate the accuracy of the NLR in predicting survival outcomes.

**Results:**

During a median follow-up of 91 months (interquartile range, 55–131 months), 896 (27.5%) of the 3251 diabetes patients died, including 261 (8.0%) with cardiovascular deaths and 635 (19.5%) with noncardiovascular deaths. The RCS regression analysis showed a positive linear association between the NLR and all-cause and cardiovascular mortality (both *p* > 0.05 for nonlinearity) in diabetes patients. Participants were divided into higher (> 3.48) and lower (≤ 3.48) NLR groups according to the MSRSM. In the multivariable-adjusted model, compared with participants with a lower NLR, those with a higher NLR had a significantly higher risk of both all-cause (HR 2.03, 95% confidence interval (CI) 1.64–2.51, *p* < 0.0001) and cardiovascular mortality (HR 2.76, 95% CI 1.84–4.14, *p* < 0.0001). The association was consistent in subgroup analyses based on age, sex, smoking status, drinking status, and hypertension, with no significant interaction between the aforementioned characteristics and the NLR (*p* interaction > 0.05). The time-dependent ROC curve showed that the areas under the curve of the 1-, 3-, 5-, and 10-year survival rates were 0.72, 0.66, 0.64, and 0.64 for all-cause mortality and 0.69, 0.71, 0.69 and 0.65, respectively, for cardiovascular mortality.

**Conclusion:**

An elevated NLR is independently associated with increased all-cause and cardiovascular mortality in diabetes patients.

**Supplementary Information:**

The online version contains supplementary material available at 10.1186/s12933-023-01998-y.

## Background

Diabetes has become a serious global public health problem over the past several decades [1, 2]. According to the 10th edition of the International Diabetes Federation (IDF) Diabetes Atlas, the number of global diabetes cases in individuals aged 20–79 years was estimated to be 536.6 million (10.5%) in 2021 and is predicted to increase to 783.2 million (12.2%) by 2045 [1]. Diabetes has been reported to be associated with an increased risk for several complications, including cardiovascular disease, nephropathy, retinopathy and neuropathy [3–5]. Furthermore, individuals with diabetes have a significantly increased risk of all-cause and cardiovascular mortality [6]. Hence, it is important to identify more risk factors in a timely manner for the prevention, delay or reduction of diabetes progression and diabetes-related death.

An ideal prognostic scoring system should not only provide independent prognostic parameters that are easily identifiable during diagnosis but also have a low cost in clinical practice. The neutrophil–lymphocyte ratio (NLR), an easily measurable parameter of global inflammatory burden and an integrated reflection of two different yet complementary immune pathways of innate (neutrophils) and adaptive (lymphocytes) cellular immune responses, has been studied as a factor correlated with disease severity and prognosis in many malignant and benign diseases [7–11]. Recently, a higher NLR was confirmed to be significantly associated with higher glycemia [12] and elevated HbA1c levels [13] in patients with diabetes. Other published studies found that an increased NLR was a risk factor for diabetic kidney disease [14, 15] and cardiovascular disease [16, 17] in diabetes patients. The NLR also emerged as the strongest predictor of the incident risk of cardiovascular events and death in a randomized clinical trial (CANTOS) including 10,061 patients with prior myocardial infarction [18]. However, the relationship between the NLR and mortality risk has not been clearly demonstrated in diabetes patients.

Therefore, we conducted this study to investigate the relationship of the NLR with all-cause and cardiovascular mortality risk in a large, nationally representative sample of diabetes patients.

## Methods

### Study population

The National Health and Nutrition Examination Survey (NHANES), a large cross-sectional research program conducted by the Centers for Disease Control (CDC) and Prevention of the USA, is designed to assess the health status in a population selected to be representative of American populations after survey weighting by using interview, examination, dietary and laboratory data. The original survey protocol was approved by the Institutional Review Board of the National Center of Heath Statistics. All participants signed informed consent forms. The present study was deemed exempt by the Institutional Review Board of our center.

**Fig. 1:**
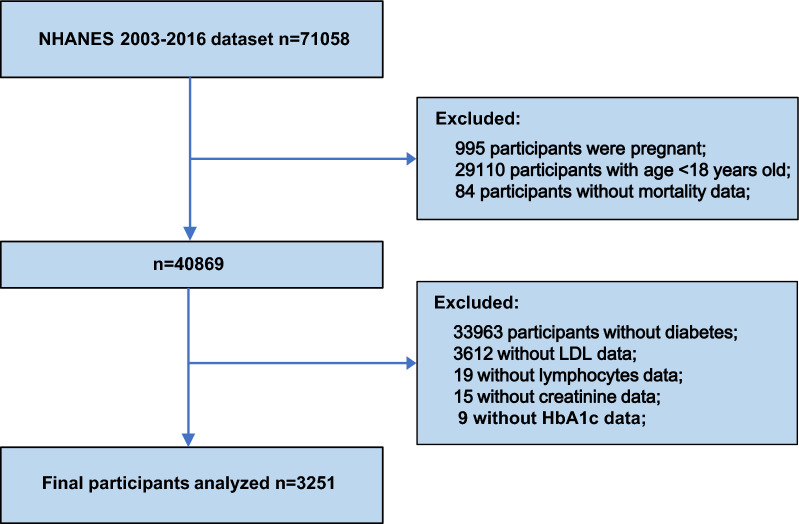
The flow chart of participants inclusion and exclusion in current study

Data for this research were taken from seven cycles of NHANES (2003–2016) with a total of 3251 participants (Fig. 1). We enrolled eligible participants with diabetes aged ≥ 18 years. Participants without complete survival and laboratory test information or who were pregnant were excluded.

### Definition of diabetes and the measurement of blood cell counts

Individuals meeting one or more of the following criteria were considered to have diabetes: (1) fasting plasma glucose ≥ 7.0 mmol/L or 2-h oral glucose tolerance test level of ≥ 11.1 mmol/L; (2) random blood glucose ≥ 11.1 mmol/L; (3) glycohemoglobin (HbA1c) ≥ 6.5%; (4) use of diabetes medication or insulin; and (5) self-reported doctor diagnosis of diabetes [19]. The complete blood count is a routine blood test used to evaluate the participant’s overall health and detect a wide range of disorders. The methods used to derive the complete blood count are based on the Beckman Coulter methodology. The NLR was calculated by dividing the neutrophil count by the lymphocyte count.

### Mortality outcomes of the study population

The source of mortality information was extracted from the National Death Index (NDI) database (https://www.cdc.gov/nchs/data-linkage/mortality-public.htm) of the CDC. The follow-up time for each individual was adopted from the time of participation to the date of death or until December 31, 2019 (the last update date of the NDI database). The International Statistical Classification of Diseases, 10th Revision (ICD-10) codes were used to identify cardiovascular deaths (I00-I09, I11, I13 and I20-I51) [20].

### Covariates

Age, sex, race, BMI, smoking status, drinking status and hypertension were adopted from demographic and health questionnaires of the NHANES survey. Age was a continuous variable, and sex and race were categorical variables. Race was classified as non-Hispanic Black, non-Hispanic White, Mexican American and others. Body mass index (BMI) was calculated as weight in kilograms divided by height (m) squared and was categorized as normal (< 25 kg/m^2^), overweight (25 ≤ BMI < 30 kg/m^2^), and obese (≥ 30 kg/m^2^). Smoking status was categorized as never a smoker (defined as smoking less than 100 cigarettes in life), former smoker (smoking more than 100 cigarettes but smoking not at all now) and current smoker (smoking more than 100 cigarettes and smoking some days or every day now) [21]. Drinking status was divided into never drinking (defined as less than 12 drinks in life), former drinking (defined as ≥ 12 drinks in 1 year and did not drink last year, or did not drink last year but drank ≥ 12 drinks in life), mild drinking (defined as ≤ 1 drink per day in females and ≤ 2 drinks per day in males on average over the past 12 months), moderate drinking (1–3 drinks per day for females and 2–4 drinks per day for males on average over the past 12 months), and heavy drinking (≥ 4 drinks per day for women or ≥ 5 drinks per day for men on average over the past 12 months) [22]. Hypertension was defined as a self-reported history of hypertension, the use of antihypertensive medication, an average systolic blood pressure ≥ 140 mmHg and/or an average diastolic blood pressure ≥ 90 mmHg. High-density lipoprotein cholesterol (HDL), low-density lipoprotein cholesterol (LDL), total cholesterol (TC), triglyceride (TG), serum creatinine (Scr), blood urea nitrogen (BUN), and HbA1c levels and blood cell counts were obtained from laboratory test results. The Chronic Kidney Disease Epidemiology Collaboration (CKD-EPI) Scr equation was used to calculate the estimated glomerular filtration rate (eGFR) [23]. The family income-to-poverty ratio was categorized as ≤ 1.0%, between > 1 and ≤ 3.0%, or > 3.0%. Education level was classified as college or above, high school or equivalent, or less than high school.

### Statistical analysis

According to the NHANES analytic and reporting guidelines [24], complex sampling designs and sampling weights were considered during analysis. The sampling weight was calculated as follows: fasting subsample 14-year mobile examination center (MEC) weight = fasting subsample 2-year MEC weight/7. Continuous variables and categorical variables were described as weighted means and percentages, respectively. For continuous variables, the Student* t* test and the Mann‒Whitney *U* test, as appropriate, were used to compare differences between two groups. For categorical data, differences between groups were evaluated by the chi-square test.

The optimal NLR cutoff point corresponding to the most significant association with survival outcomes was obtained by maximally selected rank statistics based on the ‘maxstat’ package (https://CRAN.R-project.org/package=maxstat) [25, 26], which were then used to separate participants into higher- and lower-NLR groups. Restricted cubic spline (RCS) with three knots was adopted to visualize the potentially nonlinear association between the NLR and all-cause mortality and cardiovascular mortality in diabetes patients. The association of the NLR with all-cause and cardiovascular mortality among patients with diabetes was assessed by survey-weighted Cox regression analysis. Two models were constructed to adjust for possible confounding factors. Model 1 was adjusted for age, sex, race, BMI, smoking status and drinking status. Model 2 was additionally adjusted for age, sex, race, BMI, smoking status, drinking status, hypertension, HDL, LDL, TG, TC, HbA1c, education level, the family income-to-poverty ratio and the eGFR. The probabilities of survival outcomes were calculated according to the Kaplan‒Meier method and compared using the log-rank test. The association of NLR values with mortality was analyzed by using subgroups based on age, sex, smoking status, drinking status and hypertension, and their interactions were explored. Considering the interrelationship between C-reactive protein (CRP) levels and inflammation status, we further adjusted for CRP levels in a subgroup of the study patients (CRP data was only available in the NHANES 2003–2010, N = 1735) (Additional file 1: Fig. S1 and Additional file 3: Table S1). Time-dependent receiver-operator characteristic curve (ROC) analysis [27] was conducted to evaluate the accuracy of the NLR at different time points in predicting survival outcomes by using the ‘timeROC’ package. Data were analyzed using R Statistical Software, version 4.1.0 (http://www.r-project.org). A two-tailed *p* < 0.05 indicated statistical significance.

## Results

### Characteristics of the study population

**Fig. 2:**
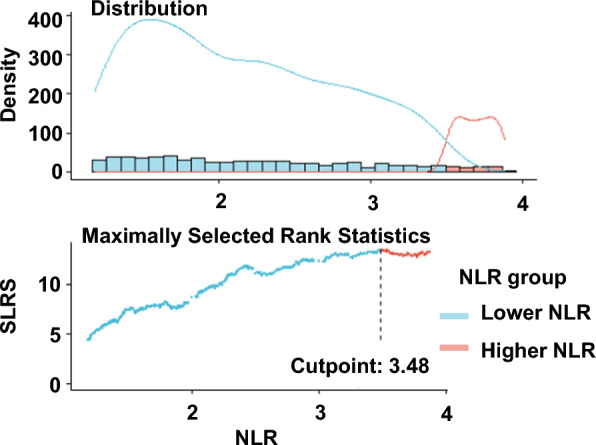
The cutoff point was calculated using the maximally selected rank statistics based on the ‘maxstat’ package. SLRS indicates Standardized Log-Rank Statistic

A total of 3251 participants with diabetes were enrolled in the present study, representing 14,691,193 patients with diabetes in the US. Using the optimal NLR cutoff value (3.48) corresponding to the most significant association with survival adopted based on maximally selected rank statistics, participants were categorized into the higher group (NLR > 3.48, n = 467) and the lower group (NLR ≤ 3.48, n = 2784) (Fig. 2). In comparison with those in the lower NLR group, the participants in the higher NLR group were older; had a higher proportion of white race; had a lower lymphocyte count, HbA1c, LDL, TG, TC and eGFR; and had a higher white blood cell count, neutrophil count, serum creatinine, BUN and HDL. More characteristics of the participants are shown in Table 1.

**Table 1:** Characteristic of participants

Variable	Total (n = 3251)	Lower NLR (n = 2784)	Higher NLR (n = 467)	*P* value
Age, years	59.59 (58.94,60.25)	58.64 (57.95,59.34)	64.62 (63.23,66.00)	< 0.0001
Sex, %				0.11
Female	49.44 (45.38,53.51)	50.36 (47.76,52.95)	44.61 (38.39,50.84)	
Male	50.56 (45.90,55.22)	49.64 (47.05,52.24)	55.39 (49.16,61.61)	
Race, %				< 0.0001
Non-Hispanic Black	13.19 (11.47,14.92)	14.63 (12.39,16.87)	5.59 (3.63,7.56)	
Mexican American	8.96 (7.19,10.73)	9.82 (7.73,11.90)	4.44 (2.16,6.71)	
Non-Hispanic White	65.54 (57.71,73.37)	62.49 (58.58,66.40)	81.67 (77.21,86.14)	
Others	12.30 (10.52,14.09)	13.06 (10.99,15.14)	8.30 (5.92,10.67)	
BMI, kg/m^2^	32.29 (31.92,32.65)	32.45 (32.10,32.80)	31.43 (30.36,32.49)	0.06
BMI category, %				0.03
Normal weight (< 25)	13.87 (11.98,15.76)	13.34 (11.59,15.09)	18.43 (14.04,22.81)	
Over weight (25–30)	27.93 (25.07,30.79)	27.95 (25.75,30.14)	31.35 (24.61,38.09)	
Obesity (≥ 30)	56.24 (51.32,61.17)	58.71 (56.16,61.26)	50.22 (43.76,56.68)	
Smoking status, %				0.09
Never	49.63 (46.00,53.27)	50.95 (48.28,53.62)	43.64 (36.98,50.30)	
Former	33.02 (29.15,36.89)	31.99 (29.47,34.51)	39.07 (32.63,45.51)	
Current	17.04 (14.91,19.18)	17.06 (15.07,19.05)	17.29 (12.71,21.87)	
Drinking status, %				0.61
Never	15.89 (13.85,17.92)	17.09 (15.00,19.18)	16.64 (12.26,21.03)	
Former	23.65 (20.76,26.53)	24.80 (22.44,27.17)	28.16 (22.60,33.72)	
Mild	30.80 (27.26,34.33)	32.78 (29.79,35.76)	34.15 (27.47,40.83)	
Moderate	11.07 (9.05,13.09)	12.13 (9.88,14.39)	10.40 (6.39,14.41)	
Heavy	11.94 (10.09,13.79)	13.20 (11.36,15.03)	10.64 (7.04,14.25)	
Hypertension, %				0.06
No	31.37 (27.86,34.88)	32.38 (29.66,35.10)	26.08 (20.29,31.87)	
Yes	68.60 (63.01,74.19)	67.62 (64.90,70.34)	73.92 (68.13,79.71)	
NLR	2.49 (2.43,2.55)	2.05 (2.02,2.08)	4.80 (4.61,4.98)	< 0.0001
WBC, × 10^9^/L	7.37 (7.27,7.47)	7.14 (7.04,7.24)	8.60 (8.23,8.96)	< 0.0001
Neutrophil, × 10^9^/L	4.51 (4.43,4.59)	4.17 (4.10,4.24)	6.34 (6.04,6.63)	< 0.0001
Lymphocyte, × 10^9^/L	2.03 (1.99,2.06)	2.15 (2.11,2.18)	1.39 (1.33,1.45)	< 0.0001
Platelet, × 10^9^/L	243.04 (239.66,246.42)	244.05 (240.43,247.67)	237.74 (227.51,247.97)	0.27
Scr, umol/L	85.24 (82.92,87.57)	83.07 (80.78, 85.36)	96.73 (89.91,103.55)	< 0.001
BUN, mmol/L	5.58 (5.48,5.69)	5.39 (5.28,5.50)	6.62 (6.28,6.96)	< 0.0001
eGFR, mL/min/1.73m^2^	84.03 (82.97,85.09)	85.80 (84.63,86.97)	74.67 (72.20,77.13)	< 0.0001
HbA1c, %	6.91 (6.84,6.98)	6.96 (6.88,7.04)	6.64 (6.45,6.83)	0.003
HDL, mmol/L	1.30 (1.28,1.32)	1.29 (1.27,1.31)	1.37(1.28,1.46)	0.1
LDL, mmol/L	2.77 (2.73,2.82)	2.80 (2.75,2.85)	2.61 (2.52,2.71)	0.001
TG, mmol/L	1.67 (1.62,1.72)	1.71 (1.66,1.76)	1.46 (1.38,1.54)	< 0.0001
TC, mmol/L	4.84 (4.78,4.89)	4.87 (4.82,4.93)	4.65 (4.51,4.80)	0.005
Education levels, %				0.47
Less than high school	10.72 (9.26,12.19)	10.82 (9.34,12.30)	10.19 (6.90,13.49)	
High school or equivalent	40.28 (35.95,44.61)	39.76 (36.56,42.95)	43.03 (36.71,49.36)	
College or above	48.94 (44.23,53.64)	49.38 (46.14,52.61)	46.60 (40.74,52.47)	
Not recorded	0.07 (0.02, 0.12)	0.05 (0.00,0.10)	0.17 (− 0.02,0.36)	
Family income-poverty ratio, %				0.85
≤ 1.0	14.47 (12.89,16.05)	14.72 (12.99,16.45)	13.15 (9.98,16.32)	
1.0–3.0	39.24 (35.16,43.32)	38.93 (36.14,41.72)	40.87 (34.80,46.94)	
> 3.0	39.03 (34.76,43.29)	39.12 (36.04,42.19)	38.57 (31.86,45.27)	
Not recorded	7.26 (5.94, 8.59)	7.23 (5.83, 8.63)	7.41 (4.79,10.04)	

Continuos variables are presented as the mean and 95% confidence interval, category variables are described as the percentage and 95% confidence interval

### Associations of the NLR with all-cause mortality

**Fig. 3:**
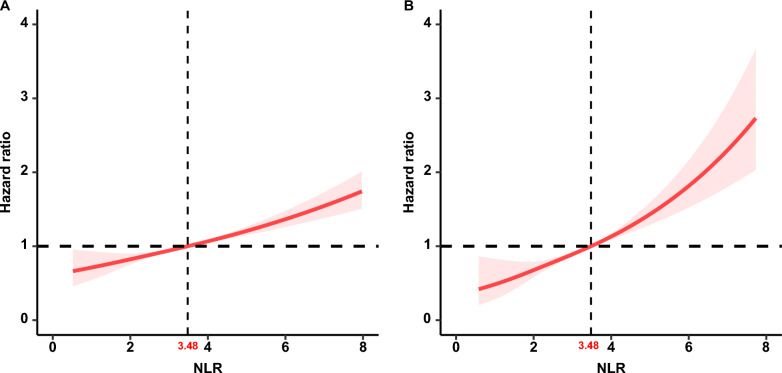
The association of NLR with all-cause (**A**) and cardiovascular mortality (**B**) among diabetes visualized by restricted cubic spline (NLR breakpoint: 3.48). Hazard ratios were adjusted for age, sex, race, BMI, smoking status, drinking status, hypertension, HDL, LDL, TG, TC, HbA1c, education level, the family income-to-poverty ratio and the eGFR. Both *p* value for nonlinearity > 0.05

**Fig. 4:**
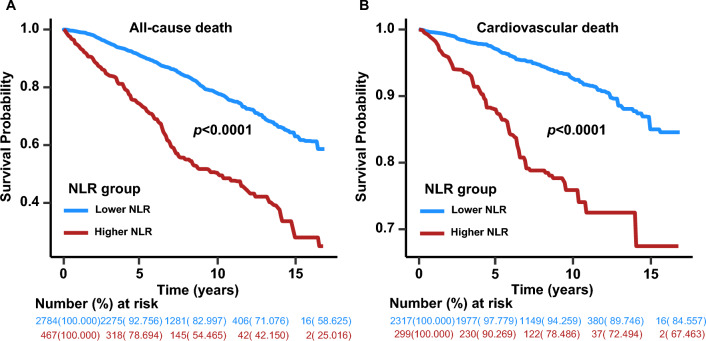
Kaplan–Meier curves of the survival rate and the number (%) of at-risk diabetes patients with higher (> 3.48) and lower (≤ 3.48) NLR values. **A** All-cause mortality. **B** cardiovascular mortality

During a median follow-up of 91 months (interquartile range (IQR), 55.5–131.0 months), 896 (27.5%) of the 3251 patients with diabetes died, including 261 (8.0%) with cardiovascular deaths and 635 (19.5%) with noncardiovascular deaths. RCS analysis showed a positive linear association between the NLR and all-cause mortality in patients with diabetes (nonlinear *p* = 0.822) (Fig. 3A). In the unadjusted model (Crude Model), we identified that the risk for all-cause mortality significantly increased as the NLR value increased (HR 1.18, 95% CI 1.12–1.24, *p* < 0.0001) (Table 2). After multivariate adjustment, each one-unit increase in the NLR value was associated with a 13% (Model 1, HR 1.13, 95% CI 1.08–1.19, *p* < 0.0001) and 14% (Model 2, HR 1.14, 95% CI 1.10–1.19, *p* < 0.0001) (Table 2) increased risk of all-cause mortality, respectively. Kaplan‒Meier survival rates for all-cause mortality differed between the higher- and lower-NLR groups (*p* < 0.0001), and the survival rate was lower in the higher-NLR group (Fig. 4A). Cox regression analysis showed that the risk for all-cause mortality significantly increased in the higher-NLR group from the crude model (HR 2.89, 95% CI 2.39–3.49, *p* < 0.0001) to the adjusted models (Models 1 and 2) (HR 2.13, 95% CI 1.75–2.60, *p* < 0.0001; HR 2.03, 95% CI 1.64–2.51, *p* < 0.0001) (Table 2).

**Table 2:** The relationships between NLR and mortality in diabetes

Characteristic	Crude model		Model 1		Model 2	
	HR (95% CI)	*p*	HR (95% CI)	*p*	HR (95% CI)	*p*
*All-cause mortality*						
NLR	1.18 (1.12,1.24)	< 0.0001	1.13 (1.08,1.19)	< 0.0001	1.14 (1.10,1.19)	< 0.0001
NLR category						
Lower NLR (n = 2784)	Ref		Ref		Ref	
Higher NLR (n = 467)	2.89 (2.39,3.49)	< 0.0001	2.13 (1.75,2.60)	< 0.0001	2.03 (1.64,2.51)	< 0.0001
*Cardiovascular mortality*						
NLR	1.21 (1.12,1.30)	< 0.0001	1.27 (1.17,1.38)	< 0.0001	1.27 (1.17,1.38)	< 0.0001
NLR category						
Lower NLR (n = 2317)	Ref		Ref		Ref	
Higher NLR (n = 299)	3.53 (2.52,4.94)	< 0.0001	2.71 (1.84,3.99)	< 0.0001	2.76 (1.84,4.14)	< 0.0001

Crude Model, unadjusted; Model 1, adjusted for age, sex, race, BMI, smoking status and drinking status; Model 2, adjusted for age, sex, race, BMI, smoking status, drinking status, hypertension, HDL, LDL, TG, TC, HbA1c, education level, family income-to-poverty ratio, and eGFR

A total of 1735 participants with diabetes with CRP data (CRP data were only available in the NHANES 2003–2010) were analyzed to determine the relationship between CRP levels and the NLR. The characteristics of these 1735 participants are shown in Additional file 3: Table S1. We found that CRP levels were weakly correlated with the NLR (R = 0.15,* p* < 0.0001) (Additional file 2: Fig. S2) by using Pearson correlation analysis. Moreover, the NLR was still an independent risk factor for all-cause mortality after adjusting for CRP levels and other confounders in the NHANES 2003–2010 (Additional file 4: Table S2).

We also investigated the association of NLR levels with all-cause mortality by using subgroup analysis based on age, sex, smoking status, drinking status and hypertension and found that the correlation of NLR levels with all-cause mortality was unchanged (Table 3). Moreover, no significant interaction between the aforementioned characteristics and the NLR was found (*p* interaction > 0.05) (Table 3).

**Table 3:** Subgroup analysis of the associations between NLR and mortality among diabetes

Characteristics	Lower NLR (≤ 3.48)	Higher NLR^#^	*p*^*#*^	*p* interaction	Higher NLR*	p*	*p* interaction
		HR (95% CI)			HR (95% CI)		
Age, y				0.704			0.085
< 60	1	2.28 (1.15–4.51)	0.018		1.96 (0.55–6.95)	0.29	
≥ 60	1	1.99 (1.58–2.51)	< 0.0001		2.97 (1.99–4.42)	< 0.0001	
Sex				0.752			0.21
Female	1	2.08 (1.45–3.00)	< 0.0001		2.54 (1.40–4.58)	0.002	
Male	1	2.03 (1.57–2.63)	< 0.0001		3.46 (2.18–5.50)	< 0.0001	
Smoking status				0.888			0.734
Never	1	2.16 (1.49–3.12)	< 0.0001		2.72 (1.49–4.99)	0.001	
Former/current	1	2.04 (1.58–2.64)	< 0.0001		3.06 (1.86–5.02)	< 0.0001	
Drinking status				0.748			0.733
Never	1	2.21 (1.28–3.80)	0.004		3.47 (1.31–9.18)	0.012	
Former/mild/moderate/heavy	1	2.03 (1.60–2.56)	< 0.0001		2.84 (1.86–4.35)	< 0.0001	
Hypertension				0.597			0.622
No	1	2.10 (1.32–3.33)	0.002		5.09 (2.11–12.32)	< 0.001	
Yes	1	2.06 (1.60–2.65)	< 0.0001		2.83 (1.88–4.27)	< 0.0001	

^#^All-cause mortality; *cardiovascular mortality. HRs were adjusted for adjusted for age, sex, race, BMI, smoking, drinking, hypertension, HDL, LDL, TG, TC, HbA1c, education level, family income-to-poverty ratio, and eGFR

### Associations of the NLR with cardiovascular mortality

A total of 2616 participants, consisting of 2317 with a lower NLR and 299 with a higher NLR, were included to calculate the associations of the NLR with cardiovascular mortality, except 635 noncardiovascular deaths. The estimated association between the NLR and cardiovascular mortality outcomes in diabetes was shown from an RCS model, and we observed that the NLR was positively linearly correlated with cardiovascular mortality (nonlinear *p* = 0.646) (Fig. 3B). Weighted multivariable Cox regression analyses also confirmed the association of the NLR with cardiovascular mortality (Table 2). In the unadjusted model (Crude model), we found that for every 1-point increase in the NLR, there was a 21% increased risk of cardiovascular mortality (HR 1.21, 95% CI 1.12–1.30, *p* < 0.0001). After adjusting for confounding factors (Models 1 and 2), the association between the NLR and cardiovascular mortality was still significant (Table 2). The Kaplan‒Meier survival plots indicated that cardiovascular mortality was higher in patients with a higher NLR than in those with a lower NLR (*p* < 0.0001) (Fig. 4B). In the crude and adjusted models (Models 1 and 2), the HRs for the higher-NLR group (NLR > 3.48) were 3.53 (95% CI 2.52–4.94) (*p* < 0.0001), 2.71 (95% CI 1.84–3.99) (*p* < 0.0001) and 2.76 (95% CI 1.84–4.14) (*p* < 0.0001), respectively, showing an elevated risk of cardiovascular mortality in the higher-NLR group (Table 2). Moreover, NLR was still an independent risk factor for cardiovascular mortality after adjusting for CRP levels and other confounders in the NHANES 2003–2010 (Additional file 4: Table S2). When subgroup analyses were conducted based on age, sex, smoking status, drinking status and hypertension, a similar association was found between the NLR and cardiovascular mortality, except in patients younger than 60 years old (Table 3). There was also no significant interaction between the aforementioned characteristics and the NLR for cardiovascular mortality (*p* interaction > 0.05).

### ROC analysis of the predictive value of the NLR for all-cause and cardiovascular mortality in diabetes

**Fig. 5:**
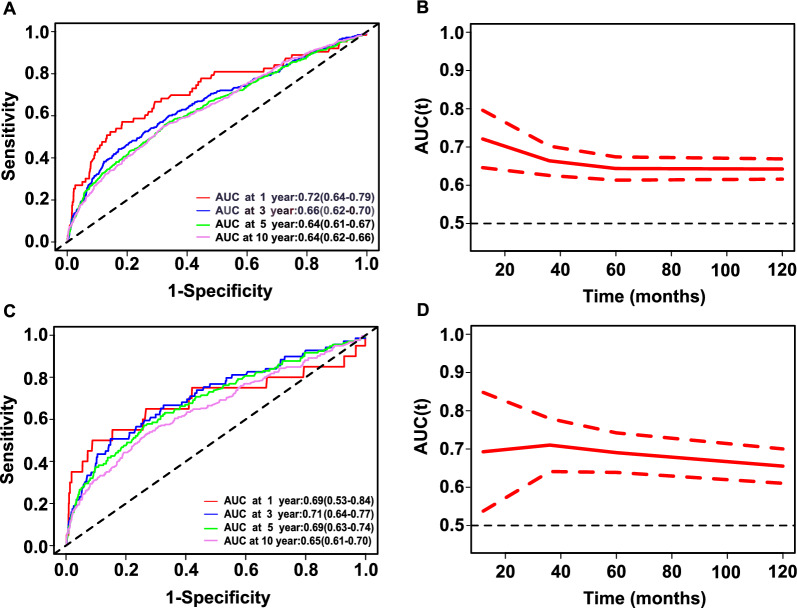
Time-dependent ROC curves and time-dependent AUC values (with 95% confidence band) of the NLR for predicting all-cause mortality (**A**, **B**) and cardiovascular mortality (**C**, **D**)

Time-dependent ROC analysis was conducted to evaluate the prognostic value of the NLR for all-cause and cardiovascular mortality in diabetes. The results showed that the area under the curve (AUC) of the NLR was 0.72 (95% CI 0.646–0.796), 0.66 (95% CI 0.625–0.702), 0.64 (95% CI 0.613–0.674), and 0.64 (95% CI 0.616–0.669) for 1-year, 3-year, 5-year and 10-year all-cause mortality, respectively (Fig. 5A and B). The AUCs of the NLR were 0.69 (95% CI 0.537–0.848), 0.71 (95% CI 0.640–0.779), 0.69 (95% CI 0.638–0.742) and 0.65 (95% CI 0.610–0.700) for 1-year, 3-year, 5-year and 10-year cardiovascular mortality, respectively (Fig. 5C and D). These results indicated that the NLR appears to have valid predictive value for all-cause and cardiovascular mortality in the short and long terms.

## Discussion

This is a large-sample study conducted to investigate the association between the NLR and survival outcomes in diabetes. In the current study, among the 3251 participants with diabetes from seven NHANES cycles (2003–2016), an elevated NLR was associated with all-cause and cardiovascular mortality and was an independent risk factor for poor survival, and these effects were only modestly attenuated after adjusting for common risk factors.

Because it consists of neutrophil and lymphocyte counts, the advantage of the NLR is that it is relatively inexpensive and ubiquitous in routine blood draws. The higher neutrophil count and lower lymphocyte count indicate an ongoing nonspecific inflammatory pathway and relatively inadequate immunity status, respectively [13]. The NLR, as an integrated marker reflecting two opposite immune pathways, is more predictive than neutrophil or lymphocyte parameters alone [9]. Previous studies indicated that activation of the immune system and chronic inflammation participate in the development of diabetes [28, 29]. It has also been proposed that the elevated NLR in diabetes probably indicates an inflammatory burden of disease [13, 29]. Moreover, recently, the NLR has been reported as a significant risk factor associated with higher glycemia [12], elevated HbA1C levels [13] and the development of diabetic kidney disease [14, 15] and cardiovascular disease [16, 17] in diabetes. These studies indicate that the NLR is expected to be a predictor for risk stratification in patients with diabetes.

Inflammatory conditions can cause numerous diabetic complications, such as diabetic nephropathy [30] and vascular complications [31]. The chronic inflammatory response in diabetes is thought to cause leukocyte recruitment to the vascular environment and contribute to endothelial damage by oxidative stress [32]. A systemic low-grade inflammatory status, as measured by high-sensitivity C-reactive protein (hs-CRP), has been reported to be an important predictor of cardiovascular complications and all-cause mortality [33]. However, the results from the ADVANCE Study showed that IL-6 levels, a circulating inflammatory marker, rather than CRP or fibrinogen levels, were an independent predictor of macrovascular events and mortality in patients with diabetes [34]. A Brazilian study based on 689 patients with diabetes reported that patients with type 2 diabetes with NLR values in the top tertile had higher incidences of major cardiovascular events, cardiovascular mortality and all-cause mortality [35]. However, the NLR cannot improve the risk discrimination in cardiovascular mortality after multivariate adjusted analyses [35]. The smaller size might blur the association between the NLR and the outcome of the event. Another possible reason is that individuals were divided into three groups according to NLR tertile, rather than calculating the optimal cutoff point based on statistical methods. Our study showed that an elevated NLR is associated with all-cause and cardiovascular mortality in 3251 diabetes patients after adjusting for confounding factors, in conditions with or without grouping by maximally selected rank statistics. The underlying mechanism might be that an increase in neutrophils can exacerbate chronic inflammation [36–38], and decreased lymphocytes contribute to a decline in immune defense, which leads to individuals’ decline in immunity and ability to resist disease [35].

An observational study including 338 patients with diabetes reported that a higher NLR (> 2.4), according to the NLR 66th percentile, was associated with higher risks of major adverse cardiac events [17]. In the present study, our results showed that diabetes patients with a higher NLR (> 3.48) experienced a poor overall survival outcome after adjusting for confounding factors (Tables 2 and 3), except for cardiovascular mortality in patients under 60 years old (Table 3). However, it is necessary to point out that the number of deaths among participants younger than 60 years old in the higher NLR group (5 deaths) is simply too small to draw conclusions. The optimal threshold (3.48) was defined by maximally selected rank statistics, which is an outcome-oriented technique providing a cutoff point that corresponds to the most significant association with survival outcomes. Moreover, according to the time-dependent ROC, the NLR also performed well in the prediction of survival, especially in the prediction of 1-year all-cause mortality (AUC 0.72) and 3-year cardiovascular mortality (AUC 0.71).

The main strength of our study is that it included a large-scale sample of individuals and a long follow-up duration, thus providing reliable conclusions and sufficient statistical power. In addition, all individuals were from the NHANES survey, thereby preventing selection bias. However, several limitations should be noted. First, even though we adjusted for several potential confounding factors in our analysis, the possibility that the NLR is affected by other unknown factors cannot be excluded. Second, this study was conducted among individuals with diabetes in the United States. Hence, whether the conclusion could be generalizable to other populations needs to be further explored.

## Conclusion

In conclusion, we analyzed 3251 participants with diabetes from seven cycles of NHANES (2003–2016) and revealed the association between the NLR and all-cause and cardiovascular mortality risks during long-term follow-up. Our findings indicate the importance of incorporating the NLR into routine clinical practice as a biomarker for predicting all-cause and cardiovascular mortality.

### Supplementary Information

**Additional file 1: Figure S1**. The flow chart of participants inclusion and exclusion in the NHANES 2003–2010.

**Additional file 2: Figure S2**. The relationship of the NLR and CRP levels in the NHANES 2003–2010.

**Additional file 3: Table S1.** Characteristic of participants in NHANES 2003–2010 (n = 1735).

**Additional file 4: Table S2.** The relationships between NLR and mortality among diabetes in NHANES 2003–2010 (n = 1735).

## Data Availability

All data analyzed during this study are publicly available on the NHANES website.

## Abbreviations

AUC: Area under the curve
BMI: Body mass index
BUN: Blood urea nitrogen
CI: Confidence interval
HbA1c: Glycosylated hemoglobin A1c
HDL: High-density lipoprotein cholesterol
LDL: Low-density lipoprotein cholesterol
NHANES: National Health and Nutrition Examination Survey
NDI: National Death Index
NLR: Neutrophil–lymphocyte ratio
RCS: Restricted cubic spline
ROC: Receiver-operator characteristic curve
TC: Total cholesterol
TG: Triglycerides
