# Supplementary figures and images for: The neutrophil–lymphocyte ratio as a risk factor for all-cause and cardiovascular mortality among individuals with diabetes: evidence from the NHANES 2003–2016
Source: Cardiovasc Diabetol. 2023 Sep 29;22:267. doi: 10.1186/s12933-023-01998-y (PMC10541705; doi:10.1186/s12933-023-01998-y)

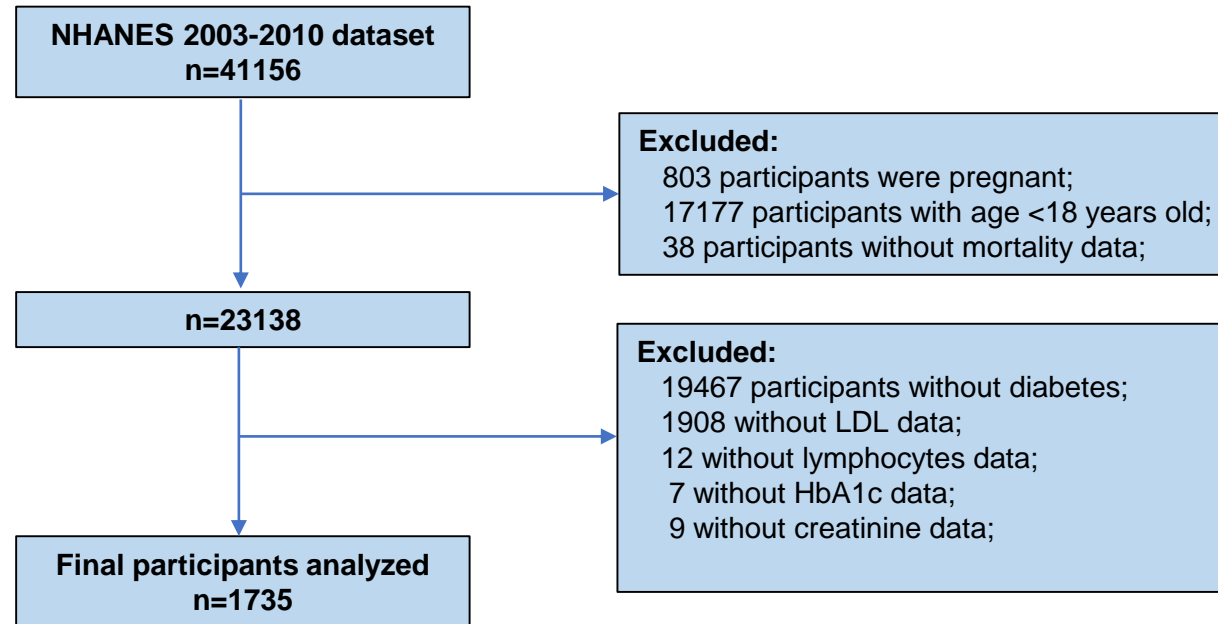

Supplement: Supplementary file 1 — Additional file 1: Figure S1. The flow chart of participants inclusion and exclusion in the NHANES 2003–2010. [file 12933_2023_1998_MOESM1_ESM.pdf]

# The relation of CRP and NLR

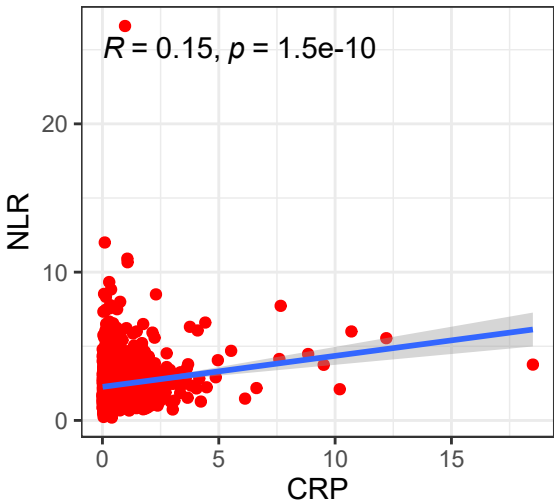

Supplement: Supplementary file 2 — Additional file 2: Figure S2. The relationship of the NLR and CRP levels in the NHANES 2003–2010. [file 12933_2023_1998_MOESM2_ESM.pdf]
